# Author Correction: Leptin receptor-expressing neuron Sh2b1 supports sympathetic nervous system and protects against obesity and metabolic disease

**DOI:** 10.1038/s41467-020-19240-8

**Published:** 2020-10-15

**Authors:** Lin Jiang, Haoran Su, Xiaoyin Wu, Hong Shen, Min-Hyun Kim, Yuan Li, Martin G. Myers, Chung Owyang, Liangyou Rui

**Affiliations:** 1 Department of Molecular & Integrative Physiology, University of Michigan Medical School, Ann Arbor, MI 48109 USA; 2 Division of Gastroenterology and Hepatology, Department of Internal Medicine, University of Michigan Medical School, Ann Arbor, MI 48109 USA; 3 Division of Metabolism and Endocrinology, Department of Internal Medicine, University of Michigan Medical School, Ann Arbor, MI 48109 USA

**Keywords:** Molecular biology, Neuroscience, Metabolic diseases, Endocrine system and metabolic diseases

Correction to: *Nature Communications* 10.1038/s41467-020-15328-3, published online 23 March 2020.

The original version of this Article contained an error in Fig. 2b, in which error bars were missing. This has been corrected in both the PDF and HTML versions of the Article.

