# Supplementary material for: Leptin receptor-expressing neuron Sh2b1 supports sympathetic nervous system and protects against obesity and metabolic disease
Source: Nat Commun. 2020 Mar 23;11:1517. doi: 10.1038/s41467-020-15328-3 (PMC7089966; doi:10.1038/s41467-020-15328-3)
Supplement: Supplementary file 1 — Supplementary Information [file 41467_2020_15328_MOESM1_ESM.pdf]

Leptin receptor-expressing neuron Sh2b1 supports sympathetic nervous system and protects against obesity and metabolic disease

Lin Jiang et al

Supplementary information

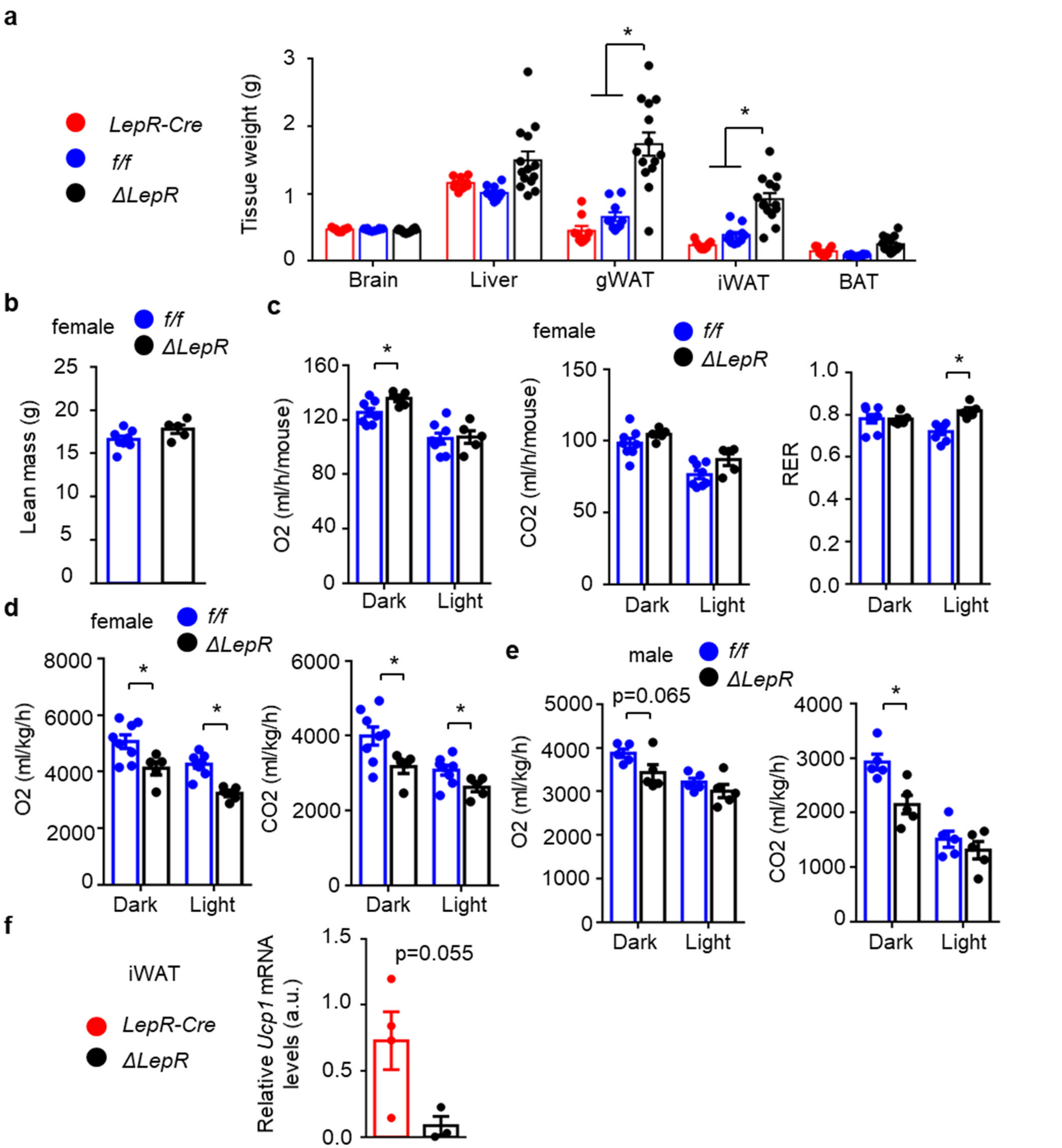

Supplementary Fig. 1. *$Sh2b1^{\Delta LepR}$* mice develop obesity. a Body compositions of males at 22 weeks of age. *$Sh2b1^{f/f}$*: n=10, *LepR-Cre*: n=9, *$Sh2b1^{\Delta LepR}$*: n=14. **b** Female lean mass at 20 weeks of age (by pDexa). *$Sh2b1^{f/f}$*: n=8, *$Sh2b1^{\Delta LepR}$*: n=5. **c-d** O_2 consumption and CO_2 production normalized to mouse (**c**) or body weight (**d**) in females at 10 weeks of age (by metabolic cages). Both **c** and **d**: *$Sh2b1^{f/f}$*: n=8, *$Sh2b1^{\Delta LepR}$*: n=5. **e** O_2 consumption and CO_2 production (normalized to body weight) in males at 10 weeks of age. *$Sh2b1^{f/f}$*: n=5, *$Sh2b1^{\Delta LepR}$*: n=7. **f** *Ucp1* mRNA in iWAT at 22 weeks of age (normalized to 36B4 levels). a.u.: arbitrary units. *LepR-Cre*: n=4, *$Sh2b1^{\Delta LepR}$*: n=3. Data are presented as mean ± SEM. *p<0.05, 1-way ANOVA/Bonferroni posttest (**a**) or 2-tailed unpaired Student's *t* test (**b-f**). Source data are provided as a Source Data file.

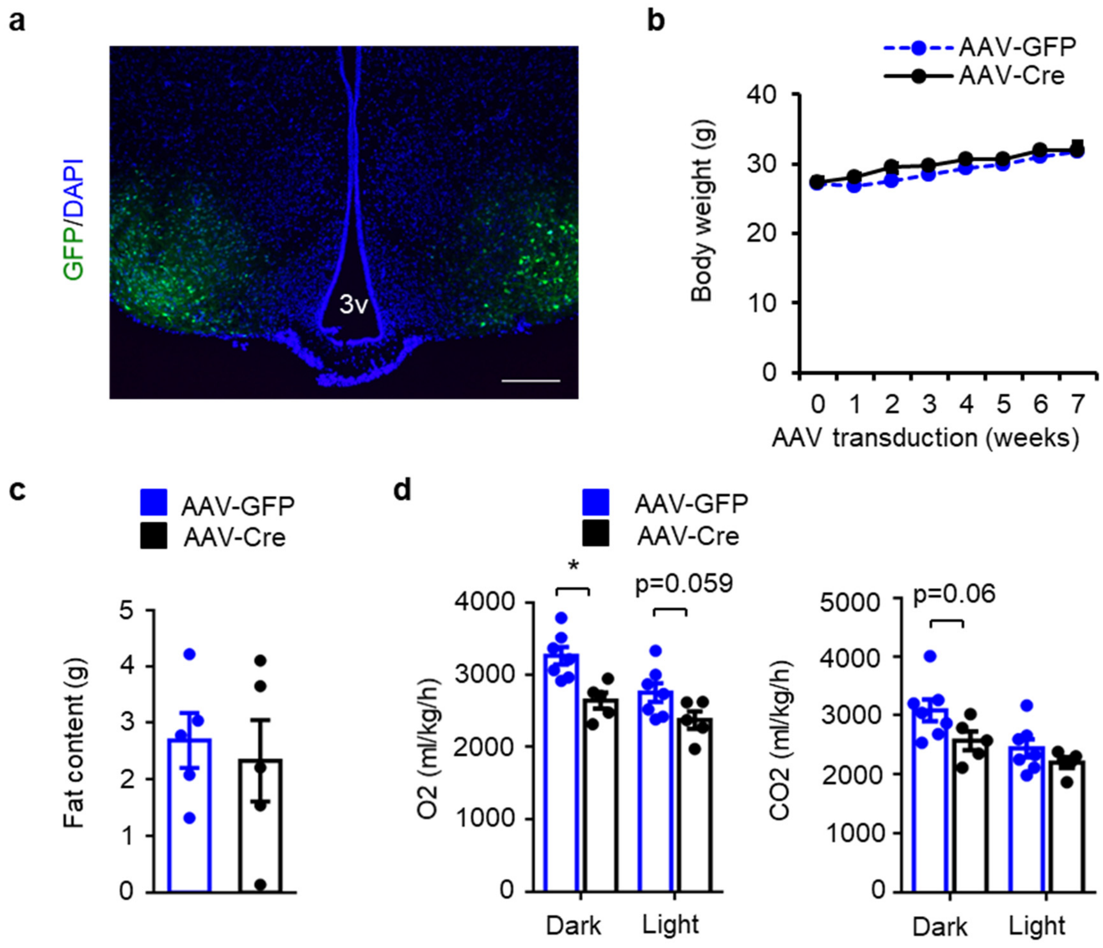

Supplementary Fig. 2. Hypothalamic expression of Cre does not alter body weight in C57BL/6 mice. a AAV9-CAG-GFP vectors were bilaterally injected into the hypothalamus. Representative hypothalamic sections (3 mice). Scale bar: 200 µm. **b-c** AAV1-hSyn-Cre or AAV1-hSyn-GFP vectors were bilaterally injected into the hypothalami of C57BL/6J mice. **b** Growth curves (n=5 per group). **c** Fat content in 7 weeks post AAV transduction (n=5 per group). **d** AAV1-hSyn-Cre (n=5) or AAV1-hSyn-GFP (n=7) vectors were bilaterally injected into the hypothalami of *Sh2b1*$^{f/f}$ male mice as in Fig 3. O_2 consumption and CO_2 production (normalized to body weight) in 10 weeks post AAV transduction. Data are presented as mean ± SEM. *p<0.05, 2-tailed unpaired Student's *t* test. Source data are provided as a Source Data file.

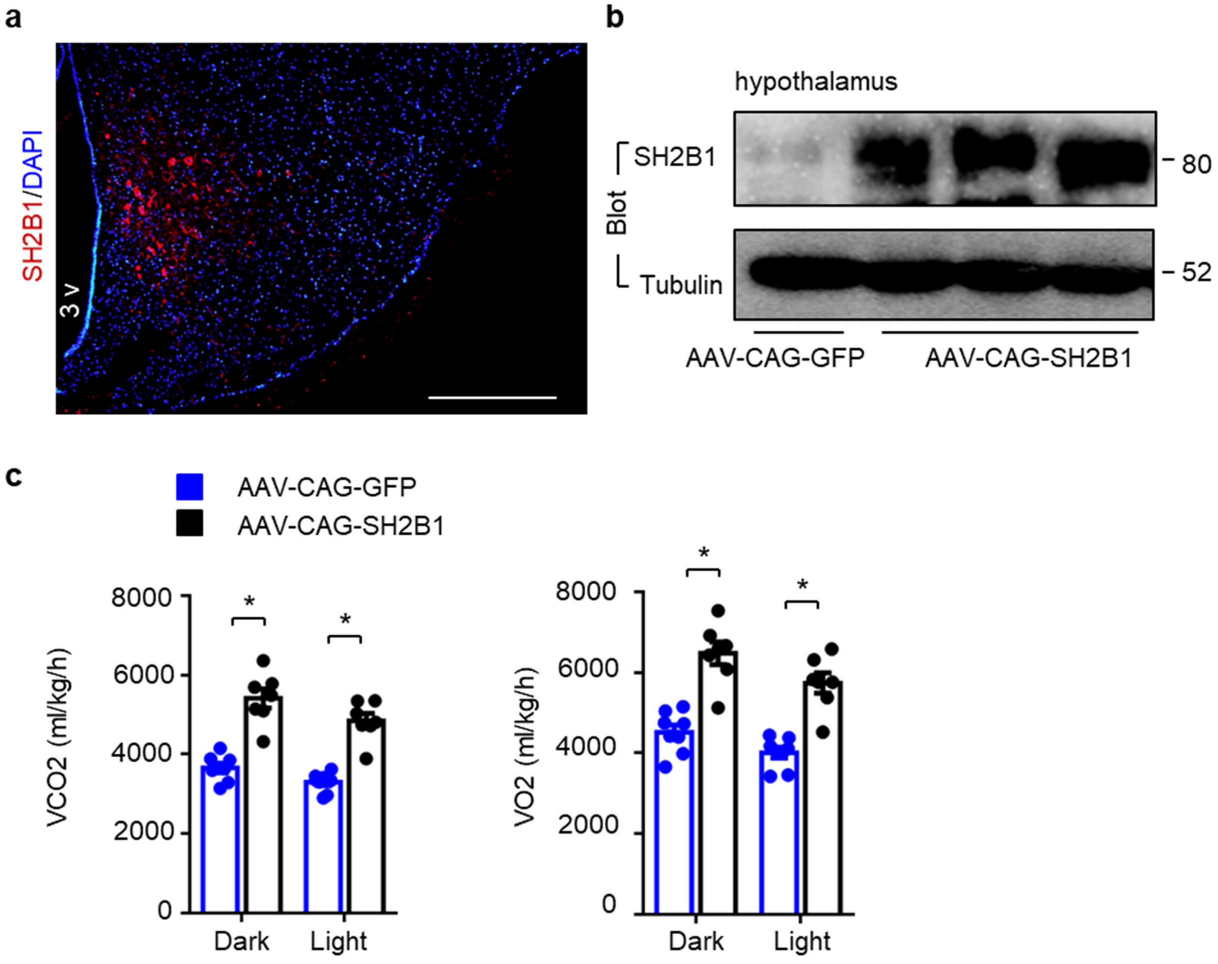

Supplementary Fig. 3. AAV9-CAG-SH2B1 vector-mediated overexpression of SH2B1 in the hypothalamus. AAV9-CAG-SH2B1β or AAV9-CAG-GFP vectors were bilaterally injected into the hypothalami of C57BL/6J male mice. **a** Representative hypothalamic sections stained with anti-Sh2b1 antibody (3 mice). Scale bar: 200 µm. **b** Hypothalamic extracts were immunoblotted with antibodies against Sh2b1 or α-tubulin. Each lane represents an individual mouse. **c** AAV vector-transduced mice were fed a HFD for 10 weeks. O_2 consumption and CO_2 production (normalized to body weight) were assessed using metabolic cages. AAV9-CAG-SH2B1β: n=7, AAV9-CAG-GFP: n=8. Data are presented as mean ± SEM. *p<0.05, 2-tailed unpaired Student's *t* test. Source data are provided as a Source Data file.

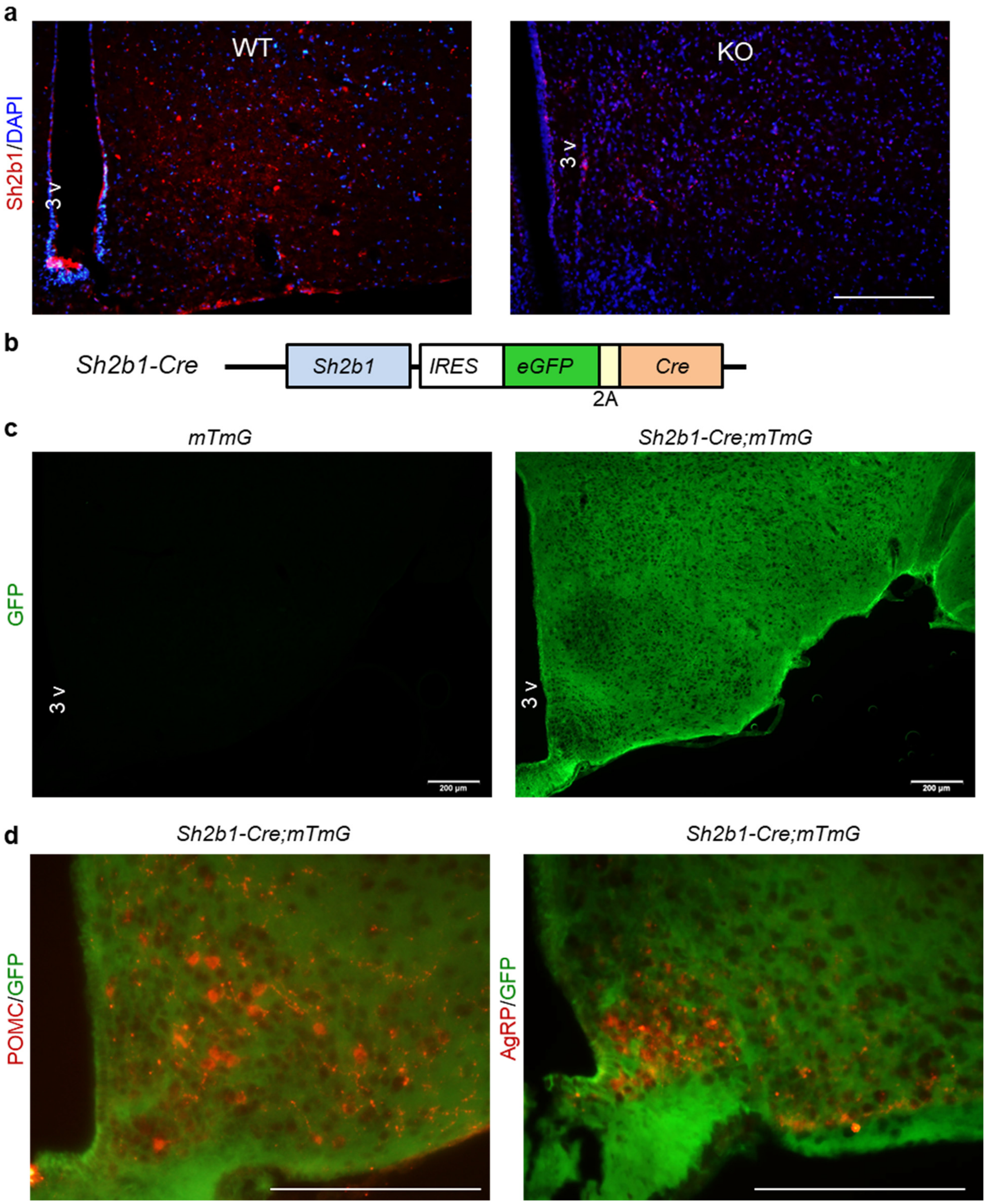

Supplementary Fig. 4. Sh2b1 neurons in mouse brains. a Representative brain sections (3 pairs). Brain sections were prepared from wild type (WT) and global *Sh2b1* knockout (KO) mice and stained with anti-Sh2b1 antibody. **b** Schematic representation of the *Sh2b1-IRES-eGFP-2A-Cre* allele. **c** Representative brain sections from *mTmG* (negative control, n=3) and *Sh2b1-Cre;mTmG* mice (20x, n=3). **d** Brain sections were prepared from *Sh2b1-Cre;mTmG* mice (n=3) and stained with anti-POMC and anti-AgRP antibodies (40x). Scale bar: 200 µm. Source data are provided as a Source Data file.

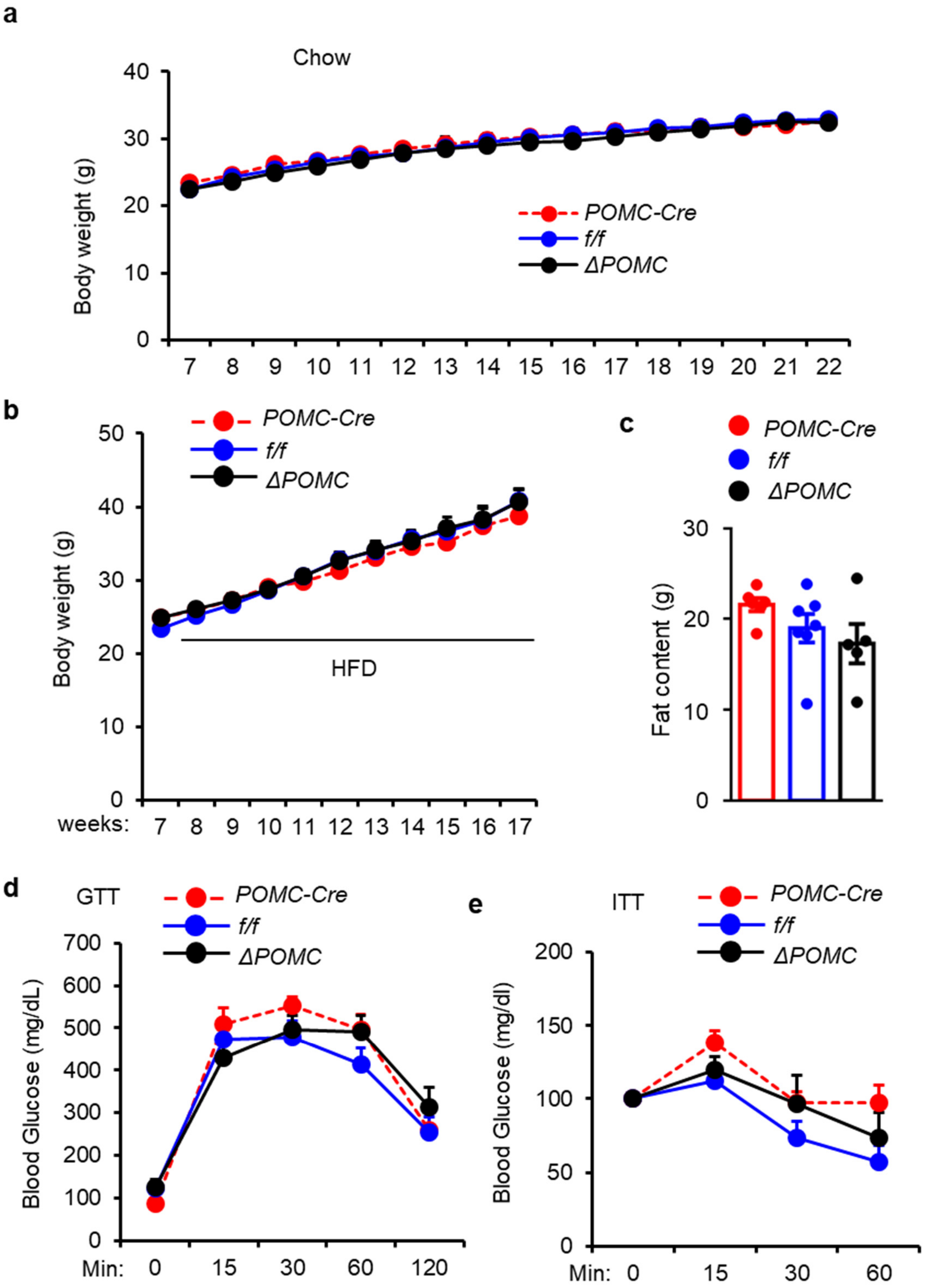

Supplementary Fig. 5. Deletion of *Sh2b1* in POMC neurons does not alter body weight and glucose metabolism. Male mice were fed a chow diet (**a**) or HFD (**b-e**). **a** Growth curves on chow diet. *Sh2b1*$^{\Delta POMC}$: n=11, *Sh2b1*$^{f/f}$: n=15, *POMC-Cre*: n=11. **b** Growth curves on HFD. *Sh2b1*$^{\Delta POMC}$: n=15, *Sh2b1*$^{f/f}$: n=7, *POMC-Cre*: n=6. **c** Fat content in 15 weeks post HFD feeding. *Sh2b1*$^{\Delta POMC}$: n=5, *Sh2b1*$^{f/f}$: n=7, *POMC-Cre*: n=6. **d-e** GTT and ITT in 15 weeks post HFD feeding. *Sh2b1*$^{\Delta POMC}$: n=9, *Sh2b1*$^{f/f}$: n=9, *POMC-Cre*: n=6. Data are presented as mean ± SEM. 1-way (**c**) or 2-way (**a**, **b**, **d**, **e**) ANAVO. Source data are provided as a Source Data file.

ANTIBODY	SOURCE	Cat#	lot#	clone#	DILUTION
Ucp-1	EMD Millipore	662045			1:10,000
Akt	Cell Signaling Technology	#4056			1:15,000
Tyrosine hydroxylase	Cell Signaling Technology	sc-14007			1:500
p-HSL (Ser660)	Cell Signaling Technology	4126			1:2,000
HSL	Cell Signaling Technology	4107			1:1,000
c-Fos	Cell Signaling Technology	2250			1:1,000
pAkt (pThr308)	Cell Signaling Technology	#4056			1:5,000
pAkt (pSer473)	Cell Signaling Technology	#4060			1:10,000
pStat3 (pTyr705)	Santa Cruz	sc-8059			1:5,000
Stat3	Santa Cruz	sc-8019			1:5,000
a-Tubulin	Santa Cruz	sc-5286			1:5,000
pHSL (pSer563)	Cell Signaling Technology	4139			1:100
p85	home made	N/A			1:10,000
TuJI (beta tubulin III)	Sigma	T8578			1:200
POMC	Phoenix Pharmaceuticals, Inc.57	H-003-57			1:2000
AgRP	Phoenix Pharmaceuticals, Inc.	H-029-30			1:2000
Sh2b1	home made				1:50

Supplementary Table 1. Antibody list.
