# Supplementary material for: Leptin receptor-expressing neuron Sh2b1 supports sympathetic nervous system and protects against obesity and metabolic disease
Source: Nat Commun. 2020 Mar 23;11:1517. doi: 10.1038/s41467-020-15328-3 (PMC7089966; doi:10.1038/s41467-020-15328-3)
Supplement: Supplementary file 3 — Reporting Summary [file 41467_2020_15328_MOESM3_ESM.pdf]

nature research

Corresponding author(s): Liangyou Rui

Last updated by author(s): 2/14/2020

Reporting Summary

Nature Research wishes to improve the reproducibility of the work that we publish. This form provides structure for consistency and transparency in reporting. For further information on Nature Research policies, see Authors & Referees and the Editorial Policy Checklist .

Statistics

For all statistical analyses, confirm that the following items are present in the figure legend, table legend, main text, or Methods section.

n/a	Confirmed	
☐	☒	The exact sample size (*n*) for each experimental group/condition, given as a discrete number and unit of measurement
☐	☒	A statement on whether measurements were taken from distinct samples or whether the same sample was measured repeatedly
☐	☒	The statistical test(s) used AND whether they are one- or two-sided<br>*Only common tests should be described solely by name; describe more complex techniques in the Methods section.*
☒	☐	A description of all covariates tested
☒	☐	A description of any assumptions or corrections, such as tests of normality and adjustment for multiple comparisons
☒	☐	A full description of the statistical parameters including central tendency (e.g. means) or other basic estimates (e.g. regression coefficient) AND variation (e.g. standard deviation) or associated estimates of uncertainty (e.g. confidence intervals)
☐	☒	For null hypothesis testing, the test statistic (e.g. *F*, *t*, *r*) with confidence intervals, effect sizes, degrees of freedom and *P* value noted<br>*Give P values as exact values whenever suitable.*
☒	☐	For Bayesian analysis, information on the choice of priors and Markov chain Monte Carlo settings
☒	☐	For hierarchical and complex designs, identification of the appropriate level for tests and full reporting of outcomes
☒	☐	Estimates of effect sizes (e.g. Cohen's *d*, Pearson's *r*), indicating how they were calculated

Our web collection on statistics for biologists contains articles on many of the points above.

Software and code

Policy information about availability of computer code

Data collection	No software and code used for data collection.
Data analysis	GraphPad Prism 6 (version 6.01), Microsoft excel 2016 (Version 1908 (Build 11929.20300))

For manuscripts utilizing custom algorithms or software that are central to the research but not yet described in published literature, software must be made available to editors/reviewers. We strongly encourage code deposition in a community repository (e.g. GitHub). See the Nature Research guidelines for submitting code & software for further information.

Data

Policy information about availability of data

All manuscripts must include a data availability statement. This statement should provide the following information, where applicable:

- Accession codes, unique identifiers, or web links for publicly available datasets
- A list of figures that have associated raw data
- A description of any restrictions on data availability

The data that support the findings of this study are available from the corresponding author upon request.

Field-specific reporting

Please select the one below that is the best fit for your research. If you are not sure, read the appropriate sections before making your selection.

☒ Life sciences ☐ Behavioural & social sciences ☐ Ecological, evolutionary & environmental sciences

For a reference copy of the document with all sections, see nature.com/documents/nr-reporting-summary-flat.pdf

Life sciences study design

All studies must disclose on these points even when the disclosure is negative.

Sample size	Sample size was calculated using N-query 6.0 with 80% power and 0.05 one-sided type I error to detect ~20% difference between mutant and control groups. Pilot studies were conducted to estimate sample size and calculate power of study.
Data exclusions	No data were excluded.
Replication	Authors confirm that data replication was successful. Experiments were conducted with calculated sample size for two or three times to ensure reproducibility. These independent experiments were combined when possible. Authors always had more than 3 samples for histology or immunohistochemistry to replicate results.
Randomization	Authors randomly allocated animals to experimental and control groups to make the experimental groups as similar as possible in all respects. In particular, the authors measured mouse body weight before the beginning of the experiment to make sure there is no weight difference between control and experimental groups.
Blinding	Investigators were blinded to analysis whenever it is available. In particular, immunohistochemistry images (Fig 5A, Fig 6G, Fig 7A and C) were quantified by non-study participants to reduce a possible bias.

Reporting for specific materials, systems and methods

We require information from authors about some types of materials, experimental systems and methods used in many studies. Here, indicate whether each material, system or method listed is relevant to your study. If you are not sure if a list item applies to your research, read the appropriate section before selecting a response.

Materials & experimental systems

n/a	Involved in the study	
☐	☒	Antibodies
☒	☐	Eukaryotic cell lines
☒	☐	Palaeontology
☐	☒	Animals and other organisms
☒	☐	Human research participants
☒	☐	Clinical data

Methods

n/a	Involved in the study	
☒	☐	ChIP-seq
☒	☐	Flow cytometry
☒	☐	MRI-based neuroimaging

Antibodies

Antibodies used	UCP-1 (EMD Millipore, 662045), Akt (CST, #4056), Tyrosine hydroxylase (Santa Cruz, sc-14007), p-HSL (Ser660) (CST, #4126), HSL (CST, #4107), c-Fos (CST, #2250), pAKT (Thr308) (CST #4056), pAKT (Ser473) (CST #4060), pSTAT3 (Santa Cruz, sc-8059), STAT3 (Santa Cruz, SC-8019), a-Tubulin (Santa Cruz, sc-5286), pHSL (Ser563) (CST #4139), TuJI (beta butulin III) (Sigma T8578), POMC (Phoenix Pharmaceuticals, H-003-57), AgRP (Phoenix Pharmaceuticals, H-029-30), Sh2b1 (generated by lab).
Validation	Manufacturer's websites state all the antibodies are verified in terms of specificity, sensitivity, and reproducibility by following methods: Analysis of a large panel of cell lines with known target expression levels, Treatment of cells with appropriate kinase-specific activators and/or inhibitors, Phosphatase treatment, Correct subcellular localization or treatment-induced translocation, Comparison of results with antibody and isotype control to ensure acceptable signal-to-background ratio, Target-specific signal verified in transfected cells, knockout cells, or siRNA-treated cells, Blocking with antigen peptide to confirm elimination of specific signal, Side-by-side comparison of a new lot with previous lots to ensure lot-to-lot consistency.

Animals and other organisms

Policy information about studies involving animals; ARRIVE guidelines recommended for reporting animal research

Laboratory animals	Authors used age-matched littermates in C57BL/6J background to improve precision and reduce variability and bias. Males and females were characterized separately to determine the potential influence of sex in data interpretations.
Wild animals	This study does not involve wild animals.
Field-collected samples	This study does not involve field-collected samples.
Ethics oversight	Animal experiments were conducted following the protocols approved by the University Committee on the Use and Care of Animals (UCUCA).

Note that full information on the approval of the study protocol must also be provided in the manuscript.
